# Effect of ultrasound-mediated blood-spinal cord barrier opening on survival and motor function in females in an amyotrophic lateral sclerosis mouse model

**DOI:** 10.1016/j.ebiom.2024.105235

**Published:** 2024-07-13

**Authors:** Anne-Sophie Montero, Ilyes Aliouat, Matthieu Ribon, Michael Canney, Lauriane Goldwirt, Samia Mourah, Félix Berriat, Christian S. Lobsiger, Pierre-François Pradat, François Salachas, Gaëlle Bruneteau, Alexandre Carpentier, Séverine Boillée

**Affiliations:** a Sorbonne Université, Neurosurgery Department, AP-HP, Pitié-Salpêtrière Hospital, Paris, France; b Advanced Surgical Research Technology Laboratory, Paris, France; c Sorbonne Université, GRC 23, Brain Machine Interface, AP-HP, Pitié-Salpêtrière Hospital, Paris, France; d Sorbonne Université, Institut du Cerveau – Paris Brain Institute – ICM, Inserm, CNRS, AP-HP, Hôpital de la Pitié-Salpêtrière, Paris, France; e Carthera, Lyon, France; f AP-HP, Pharmacology Department, Hôpital de Saint-Louis, Paris, France; g AP-HP, Centre de Reference Maladie Rare SLA, Neurology Department, Pitié-Salpêtrière Hospital, Paris, France

**Keywords:** Blood-spinal cord barrier (BSCB), Ultrasound, Amyotrophic lateral sclerosis (ALS), Motor neuron disease (MND), Lymphocytes, Neuroinflammation

## Abstract

**Background:**

Amyotrophic lateral sclerosis (ALS) is a fatal neurodegenerative disease characterized by a progressive loss of motor neurons. The limited efficacy of recent therapies in clinical development may be linked to lack of drug penetration to the affected motor neurons due to the blood-brain barrier (BBB) and blood-spinal cord barrier (BSCB).

**Methods:**

In this work, the safety and efficacy of repeated short transient opening of the BSCB by low intensity pulsed ultrasound (US, sonication) was studied in females of an ALS mouse model (B6.Cg-Tg(SOD1∗G93A)1Gur/J). The BSCB was disrupted using a 1 MHz ultrasound transducer coupled to the spinal cord, with and without injection of insulin-like growth factor 1 (IGF1), a neurotrophic factor that has previously shown efficacy in ALS models.

**Findings:**

Results in wild-type (WT) animals demonstrated that the BSCB can be safely disrupted and IGF1 concentrations significantly enhanced after a single session of transient BSCB disruption (176 ± 32 μg/g vs. 0.16 ± 0.008 μg/g, p < 0.0001). Five repeated weekly US sessions performed in female ALS mice demonstrated a survival advantage in mice treated with IGF1 and US (US IGF1) compared to treatment with IGF1 alone (176 vs. 166 days, p = 0.0038). Surprisingly, this survival advantage was also present in mice treated with US alone vs. untreated mice (178.5 vs. 166.5 days, p = 0.0061). Muscle strength did not show difference among the groups. Analysis of glial cell immunoreactivity and microglial transcriptome showing reduced cell proliferation pathways, in addition to lymphocyte infiltration, suggested that the beneficial effect of US or US IGF1 could act through immune cell modulation.

**Interpretation:**

These results show the first step towards a possible beneficial impact of transient BSCB opening for ALS therapy and suggest implication of immune cells.

**Funding:**

10.13039/501100002915Fondation pour la Recherche Médicale (FRM). Investissements d’avenirANR-10-IAIHU-06, 10.13039/501100008965Société Française de Neurochirurgie (SFNC), Fond d’étude et de Recherche du Corps Medical (FERCM), Aide à la Recherche des Maladies du Cerveau (ARMC), SLA Fondation Recherche (SLAFR), French Ministry for High Education and Research (MENR), Carthera, Laboratoire de Recherche en Technologies Chirurgicales Avancées (LRTCA).

**Research in context**

**Evidence before this study**

Amyotrophic Lateral Sclerosis (ALS) is a fast progressing invariably fatal disease with no curative treatment. For ALS, as for most neurodegenerative diseases, a major hurdle for therapies is the difficulty in getting drugs to the affected regions and as close to the affected neuronal population as possible due to the presence of the blood-brain barrier (BBB) and/or the blood-spinal cord barrier (BSCB). While some BBB impairment has been reported in ALS, the fact that active efflux transporters have been found to be upregulated can additionally limit accumulation of potentially therapeutic molecules. To increase drug penetration into the central nervous system (CNS), without damaging surrounding tissues, low-intensity pulsed ultrasound (US) in combination with intravenous microbubbles, can be used to transiently open the BBB or BSCB. The safety of this technique has recently been shown in clinical trials in patients with glioblastoma and Alzheimer's disease. In ALS, only one pilot trial has been published, in four patients, where the BBB was disrupted in a 1 cm^3^ volume of the motor cortex and was well-tolerated. However, this has not yet been adapted to target the spinal cord and no drug was co-administered to assess the impact on drug delivery.

**Added value of this study**

This study shows that US can be safely used in ALS mouse models to deliver molecules across the BSCB into the spinal cord and thus in close proximity to the affected motor neurons. Importantly, it also revealed that US, on its own, can extend survival in an ALS mouse model, potentially through beneficial changes to reactive microglial cells and increased infiltration of neuroprotective and anti-inflammatory T lymphocytes into the spinal cord.

**Implications of all the available evidence**

After demonstration of safety, US could be used to increase, and simplify repeated delivery of promising new drug therapies such as antisense oligonucleotides or antibody therapies currently being developed for ALS. For the broader ALS community, this therapeutic US strategy could provide benefits, by changing the microglial state and increasing both beneficial T cell infiltration and therapeutics into the CNS.

## Introduction

Amyotrophic lateral sclerosis (ALS) is a fatal neurodegenerative disease characterized by a progressive loss of motor neurons that leads to more than 30,000 deaths worldwide every year. The median survival after onset of symptoms is between 24 and 48 months. Currently approved treatments, such as riluzole, have very limited efficacy and prolong median survival by only two to three months.^1^

The limited efficacy of current treatments for ALS could be due to the presence of the blood-brain barrier (BBB) and the blood-spinal cord barrier (BSCB), which are obstacles to the delivery of drugs to the central nervous system (CNS).^2^^,^^3^ These barriers are formed by tight junctions of endothelial cells and astrocyte end-foot processes, with efflux transporters further limiting the penetration of most drugs.^4^ Although these barriers are partially dysregulated in ALS (endothelial cell degeneration and tight junction alteration),^5^^,^^6^ the disease-linked overexpression of active efflux transporters leads to limited additional accumulation of drugs, with riluzole brain concentrations decreased by a factor of 1.7 in ALS models compared to that in wild-type (WT) models.^7^

Low-intensity pulsed ultrasound (US) concomitant with intravenous microbubbles can be used to transiently disrupt the BBB^8^^,^^9^ and BSCB^10^^,^^11^ to enhance drug penetration without damage to the surrounding tissue. When exposed to US, microbubbles oscillate and induce mechanical stress on the vessel wall, leading to the disruption of tight junctions, nonspecific transcytosis and the downregulation of efflux transporters.^12^, ^13^, ^14^ Drug administration at the time of disruption of the BBB can lead to drug delivery enhancements of five-fold or more into the targeted tissue.^15^, ^16^, ^17^ This technique has been shown to be safe in recent clinical trials in patients with glioblastoma^18^ and Alzheimer's disease.^19^ In ALS, only one pilot trial has been published, showing the safety and feasibility of this approach using a transcranial focused US system in four patients.^20^ The BBB was disrupted over a volume of 0.35 mL in the primary motor cortex (not the spinal cord) with no drug coadministration. The procedure was well tolerated, but no efficacy data were reported.

In this study, our goal was to investigate the potential benefit of US to transiently open the BSCB in an ALS mouse model (SOD1^G93A^). US was used to repeatedly target the BSCB to induce transient disruption at the level of the lumbar spinal cord. Furthermore, the potential of combining this therapy with the coadministration of insulin-like growth factor 1 (IGF1), a known neurotrophic factor for motor neurons, was investigated. IGF1 has previously been shown to have beneficial effects in an ALS mouse model. However, this was achieved by muscle injection of a viral vector expressing IGF1 that targeted motor neurons in the spinal cord by retrograde axonal transport, thus bypassing the BSCB.^21^ Clinical trials have also been conducted with subcutaneous IGF1 delivery but showed inconsistent outcomes with limited efficacy.^22^, ^23^, ^24^ Since IGF1 has previously been shown not to cross the BBB, except in specific thalamic and hypothalamic nuclei, intranasal delivery was also tested in mice to bypass the BBB and resulted in increased IGF1 CNS concentrations.^25^^,^^26^ One clinical trial in ALS used intrathecal delivery of high and low dose IGF1 and reported a slowed decline of motor function at the high dose level, but it was conducted on only nine patients.^27^

We therefore hypothesized that IGF1 would be a good candidate to combine with transient BSCB opening using US, in order to assess its impact in ALS mice. In this work, we compared the effects of treating a SOD1^G93A^ ALS mouse model with repeated weekly treatments of US alone or US IGF1 and assessed the impact on survival as well as histological markers and molecular profiles of glial and immune cells.

## Methods

### Ethics

All animal procedures (including end stage definition) were performed in accordance with the guidelines for the care and use of experimental animals of the European Union and were approved by a local animal ethics committee under protocol number 12106 (Paris CE5, protocol #12106 to the CEF/UMS28).

### Animal models and husbandry

Eighty mutant SOD1^G93A^ female ALS model mice^28^ [B6.Cg-Tg(SOD1^G93A^)1Gur/J] (stock #004435) and 120 WT control mice on a C57Bl6/J genetic background were purchased from Jackson Laboratories (USA). We chose female mice due to randomization and ethical obligation (avoiding single housed-mice often necessary if working with males). Housing conditions were as follows: temperature, 20 ± 2 °C; humidity, 55 ± 10%; light–dark cycle, 12 h–12 h; and ventilation, which entailed approximately 12 cycles per hour of filtered, nonrecycled air. Food pellets and water were available ad libitum. Moisturized food pellets were supplied when mice became weak. To avoid distress, mice were housed several in the cage with enrichments (“cardboard home/cotton pad”). ALS mice were hemizygous for a 12-kb genomic fragment encoding the human mutated *SOD1* gene under its endogenous promoter. Mice carrying the human *SOD1* transgene were identified by polymerase chain reaction (PCR) screening of tail DNA using a mouse *SOD1* forward primer (5′-GTTACATATAGGGGTTTACTTCATAATCTG-3′), a human *SOD1* forward primer (5′-CCAAGATGCTTAACTCTTGTAATCAATGGC-3′) and a mouse and human *SOD1* reverse primer (5′-CAGCAGTCACATTGCCCAGGTCTCCAACATG-3′). This approach resulted in an ∼800 bp mouse PCR product and an ∼600 bp human *SOD1* PCR product. This transgenic model expresses large amounts of mutant SOD1, and develops adult-onset neurodegeneration of spinal motor neurons and progressive paralysis leading to death.^28^

### ALS mice monitoring

SOD1^G93A^ mice were followed for signs of paralysis, weighed weekly, from the age of 7 weeks, as an objective and unbiased measure of disease course and their grip strength measured from the age of 8 weeks (Supplementary Figs. S1 and S2).

*Grip test*: grip strength was measured weekly (Bioseb, grip test; average of three consecutive measures). Mice were handled by the tail and were allowed to grab the metal grid that they caught with their four limbs, but mainly with their forelimbs and then pulled back in the horizontal plane. The force applied to the grid just before losing grip was recorded as the peak tension. The grip test score was the mean value of the 3 consecutive weakly measures of the peak tension. Grip-test measures were used until the animal was too weak to perform this test and reached a force of around 30–40 *g*. A specific hindlimb grip-test was not performed as in our hand, in this model, it became rapidly unreliable with appearance of the symptoms. Only the four limb grip-test was therefore performed.

### Clinical evaluation

**Fig. 1:**
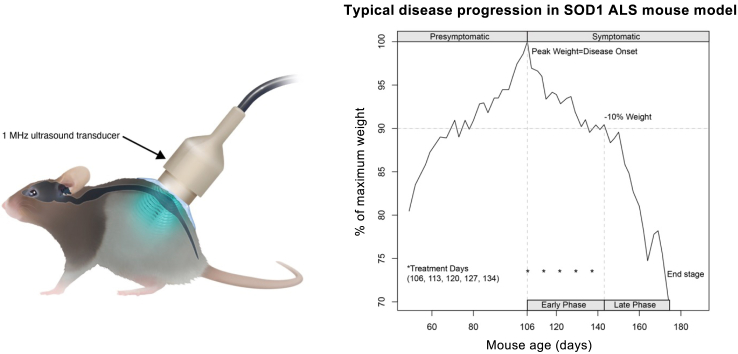
**Experimental design of low intensity pulsed ultrasound (US) mediated blood-spinal cord barrier (BSCB) opening in ALS mice.** [left] Illustration of the treatment setup. A 1 MHz ultrasound transducer was coupled to the skin with coupling gel and positioned to target the lumbar spinal cord of the mouse. BSCB disruption was performed by applying pulsed ultrasound for a duration of 150 s at time of microbubble administration. [right] Disease progression in a SOD1^G93A^ ALS mouse and the treatment protocol. Typical evolution of the weight of a mutant SOD1^G93A^ female ALS model mouse over the course of 170 days. Disease onset was defined as the time point at which the mouse was at its peak weight. Five weekly sessions of treatment with US, IGF1, or the combination of the two, were performed weekly from day 106 to day 134 for each mouse and corresponded approximately to the early phase of the disease in this SOD1 mouse model.

Weight variation is classically used as an unbiased measure of disease course in this ALS mouse model.^29^ Starting at 7 weeks, before disease onset (usually happening around the age of 106 days in this SOD1^G93A^ line) mice were weighted three times a week and then daily when they approached the 10% weight loss (usually happening around the age of 145 days in this SOD1^G93A^ line) and end stage (usually happening around the age of 166 days in this SOD1^G93A^ line). Neurological examination was performed before and after each procedure. The onset of disease in this model was defined as the time point at which the mice were at their peak weight before they started to lose weight, including, due to progressive motor neuron denervation and muscle wasting,^29^^,^^30^ as shown in Fig. 1. The following phase of early disease progression (early phase) corresponds to the phase from the peak weight to a 10% loss of maximal weight. The symptomatic stage is defined as the time at which the mouse has lost 10% of its maximal weight, has gait alterations and hindlimb splaying reflex failure. The following phase of late disease progression (late phase) corresponds to the period between the 10% weight loss and the end stage of the animal.^31^ The end stage is defined by the mouse’s incapacity to right itself up within 30 s when placed on its side; this is the endpoint used for euthanasia in mutant SOD1-expressing ALS model mice.^29^, ^30^, ^31^

### US procedure

**Fig. 2:**
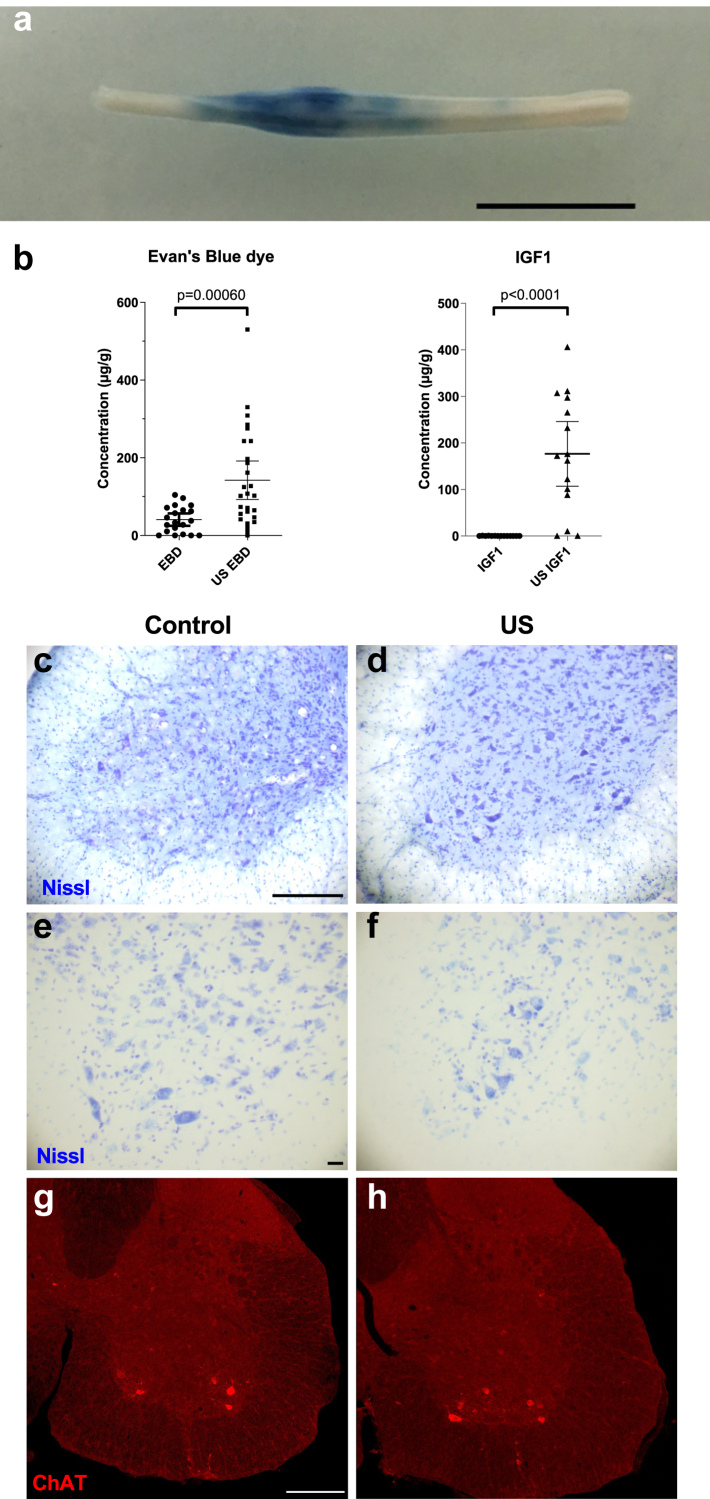
**Evans Blue Dye (EBD) and Insulin-like Growth Factor 1 (IGF1) levels were enhanced in the spinal cord after low intensity pulsed ultrasound (US) mediated blood-spinal cord barrier (BSCB) opening with no negative effect on global histological tissue integrity.** (a) Macroscopic view of the US-treated spinal cord of a wild-type mouse after EBD injection. The blue mark at the US-treated segment of the spinal cord corresponds to the local passage of the EBD into the central nervous system after BSCB opening, scale bar: 1 cm. (b) Concentration of EBD in the spinal cord of control EBD-injected (EBD, n = 20) and EBD injected and US-treated (US EBD, n = 27) wild-type mice (left). Concentration of IGF1 in the spinal cord of control IGF1-injected (IGF1, n = 14) and IGF1-injected and US-treated (US IGF1, n = 15) mice (right). The IGF1 concentration was 1100 times higher in the US-treated segments of the spinal cord, while it was only three-fold increased for EBD. This difference can be explained by the fact that IGF1 is smaller in weight (7.6 kDa) than EBD which binds with albumin forming a 68 kDa complex. Graphs represent mean with 95% Confidence interval (CI). p values were determined by Mann-Whitney test. (c, f) Images of Nissl-stained spinal cord sections of symptomatic SOD1^G93A^ mice treated (US) or not (control) with US. (g,h) Images of Choline acetyltransferase (ChAT)-immunostained spinal cord sections of end stage SOD1^G93A^ mice treated (US) or not (control) with US. Scale bars 200 μm in c for c-d, 20 μm in e for e-f, 20 μm in g for g-h.

Mice were anesthetized with an intraperitoneal mixture of ketamine (100 mg/kg) and xylazine (10 mg/kg). Body temperature was maintained using a heating pad. Back hair was shaved to minimize the amount of air trapped between the skin and US emitter. A 1-cm, 1-MHz planar US transducer was coupled to the shaved skin with US coupling gel and positioned to target the lumbar spinal cord, as shown in Fig. 1. Detailed simulations and measurements of the acoustic field of the source (planar US transducer with lateral full-width at half maximum intensity in water of 4.8 mm) have been described previously.^10^ At the beginning of US treatment, 0.2 mL of microbubbles (SonoVue, Bracco®, 8ul of hexafluoride microbubbles/ml of 0.9% sodium chloride) were injected intravenously. Ultrasound parameters were, 1 Hz pulse repetition frequency, 25 ms pulse duration, and 150 s treatment duration. Twenty-four WT mice were used to set-up the experimental protocol. The acoustic pressure used for showing efficient BSCB opening (by Evans Blue Dye (EBD) and for IGF1 assessment) in WT mice and for all ALS mouse procedures, was 0.35 MPa. No gross tissue abnormalities were detected by Nissl staining of sections in the US group compared to the control group (Fig. 2). The 0.35 MPa pressure was chosen from preliminary experiments testing a range of acoustic pressures from 0.1 to 0.85 MPa and based on our previous studies.^10^^,^^17^ The acoustic pressure was linear, so the negative and positive acoustic pressure were equivalent, and the pressure amplitude corresponded to the pressure amplitude measured in water. The acoustic pressure used for tolerance in WT mice was 0.50 MPa. Before every US treatment, we verified the ultrasound setup with the “acoustic fountain method”: when the system was turned on in water, a slight pulse was visible on the water surface every second.

### Safety and efficacy of BSCB opening in WT mice using EBD and IGF1

The neurological tolerance (measured by grip test (Supplementary Fig. S1a) and visualized by the normal gait of the mice) of five repeated BSCB opening sessions (once a week for five weeks) was assessed in 20 WT mice: 10 treated mice with US and 10 non-US-treated control mice.

The opening of the BSCB was assessed in 27 WT mice by quantifying the uptake of EBD in the spinal cord Fig. 2a–b. EBD (2% in saline solution, 4 mL/kg, 68-kDa with albumin complex) was injected intravenously (retro-orbital injection with 25G needle) just after US treatment. Mice were euthanized 30 min later with an intraperitoneal injection of pentobarbital (400 mg/kg) and were perfused transcardially with 20 mL of 1X phosphate-buffered saline (PBS) to wash out the residual intravascular EBD of the fresh tissue. The spinal cord was removed and frozen in liquid nitrogen. The amount of EBD in the homogenized sample was determined with spectrophotometry at 620 nm. The results are given in micrograms of EBD per gram of tissue. Twenty-seven US-treated segments (lumbar spinal cord) were compared to 20 non-US-treated segments (cervical spinal cord) of the same mouse. A representative example of a BSCB opening with EBD is shown in Fig. 2a.

IGF1 spinal cord uptake quantification was assessed in 29 WT mice. Mice received a subcutaneous injection of 500 μg/kg rhIGF1 (mecasermin, IPSEN Pharma®) and underwent US treatment 30 min later. This timeframe was chosen according to.^32^ Mice were euthanized 1 h later with an intraperitoneal injection of pentobarbital (400 mg/kg). For IGF1 quantification (fresh tissues), the mice were perfused transcardially with 20 mL of 1X PBS to wash out the residual intravascular IGF1. The spinal cord was removed and frozen in liquid nitrogen. IGF1 measurements were performed on spinal cord segments using the human IGF1 Quantikine enzyme-linked immunosorbent assay (ELISA) kit (R&D Systems), according to the manufacturer's instructions. The results are reported in micrograms of IGF1 per gram of protein. Fifteen US-treated lumbar spinal cords were compared to 14 non-US-treated lumbar spinal cords.

### Experimental protocol in SOD1^G93A^ ALS mice

Mutant SOD1^G93A^ ALS mice were randomly assigned to one of four treatment groups (Microsoft Excel Software): no treatment (control, n = 15 mice), US alone (US, n = 25 mice), subcutaneous injections of IGF1 alone (IGF1, n = 15 mice), or US and subcutaneous IGF1 injections (US IGF1, n = 25). IGF1 and US IGF1 mice received a subcutaneous injection of 500 μg/kg rhIGF1 (mecasermin, IPSEN Pharma®, 7.6 kDa). Mice in the control and US groups received a subcutaneous injection of 0.9% saline. Mice in the US and US IGF1 groups underwent US treatment 30 min after saline or IGF1 injection.

In each group, mice were randomly assigned to be euthanized at the symptomatic stage (defined as 10% weight loss) or at disease end stage for immunohistological analysis. The 45 residual mice were followed until death (n = 10 control mice, n = 10 US mice, n = 10 IGF1 mice and n = 15 US IGF1 mice). Sample size was chosen according to guidelines of.^33^

The intervention (control, US, IGF1, and US IGF1) started for each mouse at postnatal day 106 (P106; corresponding to the usual mean age of disease onset/weight peak for this model) and was administered once a week for five consecutive weeks, as shown in Fig. 1. Mice in the control and IGF1 groups received an intravenous injection of saline instead of microbubbles and did not undergo US.

### Animal euthanasia, tissue preparation and immunohistology

Mice were anesthetized with an intraperitoneal injection of pentobarbital (400 mg/kg). For immunohistological analysis and motor neuron counting (fixed tissues), the mice were transcardially perfused with 20 mL PBS and then 50 mL of 4% paraformaldehyde in phosphate buffer. The spinal cord was extracted, placed in 4% paraformaldehyde for 4 h for postfixation and then cryopreserved in a 30% sucrose solution in PBS before freezing in isopentane.

#### Immunostaining

Thirty-micron thick transverse serial sections across the entire lumbar spinal cords were cut on a cryostat at **−**18 °C. Floating sections of spinal cord were stored in PBS solution at 4 °C. Each well contained 8–10 sections spaced out throughout the lumbar spinal cord, representative of the whole lumbar spinal cord. Immunostainings were performed on 8–10 sections/animal.

As we hypothesized that BSBC disruption can modify glial and immune cell activity, we quantified lymphocytes, microglial and astrocytic reactivity. Immunostainings was performed on n = 3 controls, n = 4 US, n = 4 IGF1, and n = 5 US IGF1 treated (mutant SOD1^G93A^) mice for the symptomatic stage, and n = 6 controls, n = 6 US, n = 4 IGF1, and n = 5 US IGF1 treated (mutant SOD1^G93A^) mice for end stage. For evaluation of lymphocytes and glial cells, sections were incubated overnight at room temperature with the following antibodies: for microglial cells: rabbit anti-Ionized calcium binding adaptor molecule 1 (Iba1, 1:500 Wako Chemicals cat # 019-19741, RRID:AB_839504), for astrocytes: anti-glial fibrillary acidic protein (GFAP, 1:3000 Dako cat# Z0334, RRID:AB_10013382), for lymphocytes: rat anti-CD4 (1:100, AbD Serotec, cat # MCA1767, RRID:AB_322769), rat anti-CD3 (1:100, AbD Serotec, cat # MCA500G, RRID:AB_321252), and for cell proliferation: mouse anti-Ki67 (1:200, BD Pharmingen, cat # 556003, RRID:AB_396287). To determine CD4+ T-cell subtypes, a sequential (since anti-CD3 and anti-CD4 antibodies were made in rat) double-staining protocol was performed in which labelling of CD4+ cells was performed first, and the staining revealed by a (goat anti-rat) Alexa Fluor-594 fluorescent secondary antibody (1:1000, Life Technologies, cat # A-11007). In a second step, all the T lymphocytes were labelled using the rat-anti CD3+ antibodies revealed by a (goat anti-rat) Alexa Fluor-488 fluorescent secondary antibody (1:1000, Life Technologies, cat # A-11006).

For SOD1 staining, a rabbit polyclonal antibody to SOD1 (1:10000, Merck Millipore cat # 07-403-I) was used. This antibody is not specific for misfolded SOD1 but recognizes aggregated SOD1 in patients with ALS carrying mutations in SOD1 and gives a diffuse staining in patients with ALS without SOD1 mutations as we have previously shown.^34^ In addition, it recognizes aggregates in spinal cords of hSOD1^G93A^ ALS mice, gives a diffuse staining in control mice carrying the wild-type human SOD1 (Jax B6.Cg-Tg(SOD1)2Gur/J cat # 002298), and only a faint staining in control C57Bl6/J mice (Supplementary Fig. S3). SOD1 staining was revealed by a (goat anti-rabbit) Alexa Fluor-594 fluorescent secondary antibody (1:1000, Life Technologies, cat # A-11037).

#### Motor neuron counts

Motor neuron counts were determined manually from 30 μm serial sections across the entire lumbar spinal cords and counted in the spinal cord ventral horn. Counts were performed on every 12th cresyl violet acetate-stained section corresponding to a total of 16–20 sections per animal at the symptomatic stage and disease end stage. Nissl-stained motor neurons were recognized by their positioning in the ventral horn spinal cord and their large size compared to other neurons (Fig. 2c–f). Motor neurons were counted when their white nucleus and dark purple nucleoli were visible. For disease end stage, motor neuron counts were also performed after Choline acetyltransferase (ChAT) immunostaining. Spinal cord sections were incubated for 3 days at 4 °C with goat anti-ChAT (1:200 Sigma–Aldrich cat # AB144P, RRID:AB_8436037123283) and with a donkey anti-goat Alexa Fluor-594 fluorescent secondary antibody (1:1000, Life Technologies, cat # A11058).

### Immunostaining analyses

Immunostaining analyses (Iba, GFAP, ChAT, SOD1 staining), of the spinal cord were performed using an Axioscan scanner (Zeiss) with Zeiss ZEN v.3 software.

#### Motor neuron diameter

Motor neuron Feret’s diameters were measured on ChAT-stained lumbar spinal cord sections and performed using Fiji software (ImageJ).

#### Microglial immunoreactivity

The Iba1-stained lumbar spinal cord section scan protocol kept the same parameters (time and intensity of exposure and focus strategy) for every spinal cord section, and only coarse and fine focuses were adapted to each slide. Fiji software was used to measure spinal cord microglial immunoreactive intensity (the entire spinal cord section was analysed) reported as immunoreactive area, as follows: a common microglial cell detection threshold was set up for every section, and the percentage area (measuring the Iba1^+^ fluorescent immunoreactivity area in the parenchyma) was calculated. Measurements were made from every 24th 30 μm-thick lumbar spinal cord section, which corresponded to a total of 8–10 sections per animal.

#### Microglial morphology

Skeleton analyses followed recommendation from^35^ on Iba1-stained lumbar spinal cord cross section using Fiji software (ImageJ). Images were converted to 8-bit grey levels, and the contrast optimised. Iba1 positive microglial cells were selected by fluorescence intensity. Pictures were cleaned by removing outliers, artefacts and single positive pixels and the ‘skeletonize’ plugin was applied. Numbers of branches per cell and total length of branches per cell were collected. All acquisitions and analyses for each individual staining were done using the same parameters for every animal to be comparable.

#### T cell counts

CD4+ or pan-T CD3+ cell numbers were determined manually. Counting was performed using an AxioImager Z1 microscope (Zeiss).

CD3, CD4 and high magnification GFAP, Iba-1, ChAT and SOD1 pictures were done with a Leica SP8 X White Light Laser Confocal microscope.

### RNA extraction and RNAseq analyses of isolated microglial cells

Microglial cells were isolated using a modified protocol based on the method previously used in.^29^^,^^36^ The whole procedure was performed at 4 °C and did not use enzymatic digestion/incubation at 37 °C to avoid ex-vivo activation of the cells. Briefly, symptomatic SOD1^G93A^ female mice (72–96 h after the 5th US treatment) were deeply anesthetized using Xylazine (Rompun 0.2%) and Ketamine (Imalgene 500) and transcardially perfused with ice-cold 0.1 M PBS. Spinal cords were flushed using 0.1 M PBS and the lumbar spinal cords were Dounce homogenized in ice cold Hank’s balanced salt solution (HBSS) 10 times with the loose and 5 times with tight pestles. Tissues were passed through a 70-μm cell strainer (Corning). Cells were pelleted by centrifugation (300 g, 5 min), resuspended in 40% Percoll solution (GE Healthcare), HBSS and centrifugated (30 min, 500 g, 4 °C). Myelin was then removed with the Percoll. Cell pellet was washed in HBSS and resuspended in 0.1 M PBS, 0.5% BSA (Sigma Aldrich), 2 mM EDTA (Life Technologies). Microglial cells were isolated using an anti-CD11b magnetic microbead-coupled antibody and MS columns (Miltenyi Biotec cat#130-049-601) according to manufacturer’s instructions. Between 12000 and 53000 microglial cells were recovered per lumbar spinal cord (one spinal cord from one animal per sample, n = 5 control and n = 5 US). Cells were centrifuged at 300 g at 4 °C lysed in RLT Plus (Qiagen) and kept at **−**80 °C until further use.

RNA was extracted using the RNeasy plus micro kit (Qiagen). RNA quality was determined with TapeStation 2200 (Agilent). Whole total eluted RNA of microglia (between 400 and 700 pg) was used to perform cDNA synthesis using the Smart-Seq v4 Ultra Low Input RNA kit for sequencing (TaKaRa) with linear pre-amplification. Library preparation was made with 150 pg of amplified cDNA for every sample using the Nextera XT DNA Library Preparation Kit (Illumina). Samples were sequenced on a NovaSeqXplus sequencing system (Illumina) with an average coverage of 2 × 50 millions of reads per sample (150 bp, paired-end) after demultiplexing. Library preparations and sequencing were performed by the ICM iGenSeq core facility.

#### RNA-Seq data analysis

Raw data was processed with Dragen Illumina DRAGEN bio-IT Platform (v3.10.11), using default parameters, for adaptor trimming, alignment to the mm10 reference genome with the gencode m25 gtf file and expression quantification. Library orientation, library composition and coverage along transcripts were checked with Picard tools. Downstream analyses were conducted with R (v4.1.1) via a shiny interface provided by the Data Analysis Core (DAC) of the Paris Brain Institute (ICM). cpm-normalization was performed with edgeR (v3.28.0)^37^ for initial filtering. Differential analysis was performed with the ‘glm framework likelihood ratio test’ from the DESeq2 (v1.34.0)^38^ workflow. Enrichment analysis was conducted with clusterProfiler R package (v4.2.2)^39^ with ‘Gene Set Enrichment Analysis' (GSEA) on the HALLMARK (MSigDB) genesets/pathways database.

### Statistics

GraphPad (GraphPad Software, San Diego, CA) Prims v10 was used for all statistical analyses. Prior to statistical analysis of the data, distribution (D’Agostino & Pearson normality test) and homogeneity (Brown-Forsythe test) were verified. Normal distributed data were analysed with parametric test e.g., classical one-way ANOVA or Student’s t-test. Non normal distributed data were analysed with non-parametric test e.g., Kruskal-Wallis or Mann-Whitney test. One-way ANOVA or Brown-Forsythe corrected ANOVA tests were followed by Šídák or Dunnet’s multiple comparison post hoc tests to compare US vs. control groups and US IGF1 vs. IGF1 groups. We have chosen to compare results from the US group to the control group and the US IGF1 group to the IGF1 group to analyse the impact of the US and since the two cohorts were produced independently.

If sample size were lower than n = 5, distribution was considered as Normal as sample size is too small to perform a robust distribution test. Statistical significance was set at p < 0.05. Data are presented as the mean ± standard deviation (SD) for descriptive data or mean with 95% confidence interval (CI) for all other continuous quantification data. Mean or median differences with 95% CI are reported in Supplementary Table S1.

EBD and IGF1 concentrations were compared using the Mann-Whitney rank comparison test. The durations of the early and late phases were compared among groups by analysis of variance (ANOVA) followed by a post hoc Šídák multiple comparison test as well as, ChAT-stained motor neurons, astrocytic immunoreactivity, microglial immunoreactivity, microglial morphology, and lymphocyte (CD4 and CD3) numbering. Brown-Forsythe corrected ANOVA (as variance are not homogenous) followed by Dunnett multiple comparison post hoc test was used to compare data from motor neuron counts after Nissl staining and motor neuron Feret’s diameter, SOD1 immunostaining, astrocytic immunoreactivity at end stage, and microglial immunoreactivity at symptomatic stage.

Survival analysis (disease onset and end stage) was performed with Kaplan-Meier statistics. A log-rank test was performed to compare all the survival curves together. Curves were then compared two by two, and p values were adjusted by Bonferroni correction.

Data that have been censored are reported in Supplementary Table S2.

### Role of funders

Carthera provided technical support for the preclinical system. The funders “Recherche en Technologies Chirurgicales Avancées: LRTCA” (to AC), “Fondation pour la Recherche Médicale: FRM” (to SB) “Aide à la Recherche des Maladies du Cerveau: ARMC” (to SB), “SLA Fondation Recherche: SLAFR” (to SB) have provided the necessary resources to be able to perform the described experiments and student’s fellowships: “Fondation pour la Recherche Medicale” (DEA20170637795 to ASM), “société française de neurochirurgie” (SFNC to IA), “fond d’étude et de recherche du corps medical” (FERCM to IA) and French Ministry of research (MESR to FB). The founders had no role in study design, data collection, data analyses, interpretation, or writing of report.

## Results

### US treatment opened the BSCB and increased concentrations of EBD and IGF1 in the spinal cord

Mice were treated using the experimental setup illustrated in Fig. 1. A 1 MHz ultrasound transducer was used to deliver US for a duration of 150 s concomitantly with systemic injection of microbubbles. To validate that US can be used to disrupt the BSCB of WT mice, we measured EBD and IGF1 concentrations in the spinal cords of WT mice after US treatments (Fig. 2a and b). EBD concentrations were 3.4 times higher in US-treated lumbar spinal cord segments than in non-US-treated cervical spinal cord segments (142 μg/g ± 125 vs. 41 μg/g ± 34, mean ± SD, p = 0.00060, Mann-Whitney). IGF1 concentrations were 1100 times higher in the US-treated regions than in the non-US-treated regions (176 ± 125 μg/g vs. 0.16 ± 0.30 μg/g, mean ± SD, p < 0.0001, Mann-Whitney), confirming that BSCB opening via US leads to higher drug concentrations in the spinal cord.

### Repeated BSCB opening by US in WT and ALS mice was well tolerated

In WT mice, body weight and motor functions were comparable between 10 mice that underwent 5 US treatments (once a week) and 10 mice that did not undergo US treatment (Supplementary Fig. S1a). In the WT mice that underwent repeated US treatments (a total of 94 procedures), two mice (1 US-treated and 1 non-US-treated) died of retro-orbital injection complications. These findings suggest that five US treatments did not result in immediate or cumulative toxicity in WT mice.

In ALS mice (mutant hSOD1^G93A^), 4 of the 50 mice (in the US or US IGF1 groups) that underwent a total of 242 BSCB opening procedures, had complications, potentially related to US treatments (1.7% of procedures, Supplementary Table S2). One mouse (in the US group) presented paraplegia after the third US treatment. One mouse (in the US IGF1 group) presented a partially regressive motor deficit of one lower limb after the third US treatment. Two mice (in the US and US IGF1 groups) presented a totally regressive motor deficit of one lower limb after the third US treatment. In addition, seven non-US-related deaths occurred due to repeated intravenous injections and general anesthesia (2.7% of procedures). Deaths were distributed among groups and were not linked to any one intervention. The US procedure was therefore well-tolerated.

At the spinal cord tissue level, no overall anatomopathological differences were seen between control and US-treated mice after Nissl staining, nor of the motor neurons themselves after Nissl staining or ChAT immunostaining (Fig. 2c–h).

### US treatment, on its own, prolonged the survival of mutant SOD1 ALS model mice

**Fig. 3:**
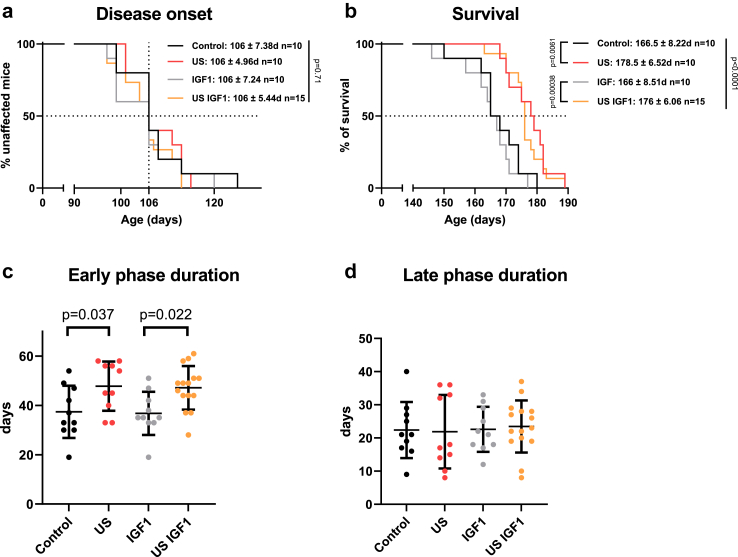
**Disruption of the blood-spinal cord barrier (BSCB) by low intensity pulsed ultrasound (US) prolongs the survival of ALS mice with or without Insulin-like Growth Factor 1 (IGF1).** (a and b) Kaplan-Meier curves for (a) the age of disease onset of the mice in the different treatment groups and (b) the survival times of the different groups of treated mice. Control: ALS mice, US: US-treated ALS mice, IGF1: IGF1-treated ALS mice, US IGF1: IGF1-treated and US-treated ALS mice. p values were determined by log-rank test to compare all groups and log-rank test with Bonferroni correction between two separate groups. (c and d) Impact of treatment on (c) early-phase disease duration (from onset to the symptomatic stage defined as 10% of weight loss) and (d) late-phase disease duration (from the symptomatic stage to end stage). Note that mice were followed from the age of 7 weeks and that the treatment was stopped at 134 days. Bars: mean ± SD. p values were determined by ANOVA followed by a post hoc Šídák multiple comparison test.

We next aimed to test whether the US treatment enhanced delivery of IGF1, a neurotrophic factor that does not normally cross the BBB or BSCB,^40^ and could increase survival in our ALS mouse model. There was no difference between all four groups in the age of mice at disease onset (Fig. 3a, median = 106 days, p = 0.71, log rank test, measured retrospectively by weight peak (Supplementary Fig. S2) and corresponding to the start of the treatment) confirming the homogeneity of the four randomized groups before treatments. Survival time of mice in the group treated with IGF1 and US (US IGF1 group) was increased compared to that of those in the group treated with IGF1 alone (median ± SD 176 ± 6.06 vs. 166 ± 8.51 days, p = 0.0038, log rank test, Fig. 3b). However, and surprisingly, mice in the US group (that did not receive IGF1) also showed a similar improvement in survival time compared to mice in the control group (median ± SD 178.5 ± 6.52 vs. 166.5 ± 8.22 days, p = 0.0061, log rank test, Fig. 3b), showing that the US treatment, by itself, already led to the full survival enhancement. Of note, we also followed muscle strength using grip test. While we measured an expected decrease with disease, there was no difference among the different groups. However, the grip test we performed, measures mainly the strength of the forelimbs, and our US treatment specifically targeted hindlimbs (Supplementary Fig. S1b and c).

The increased survival time in ALS mice that either underwent US or US and IGF1 treatments, was due to an effect on the early disease phase duration, which was prolonged in the mice of the US-treated groups compared to that of mice in the control group and in mice in the US IGF1 group compared to mice in the IGF1 group (mean ± SD, US = 47.8 ± 10, control = 37.4 ± 10.6, p = 0.037, US IGF1 = 47 ± 8.8, IGF1 = 36.8 ± 8.8, p = 0.022, ANOVA followed by a post hoc Šídák multiple comparison test, Fig. 3c). Importantly, the last treatment was 28 days after the theoretical disease onset (P135), and therefore, all treatments occurred only during the early phase of the disease. There was no difference among the groups in terms of the duration of the late phase of the disease (after the last treatment, Fig. 3d).

In summary, five repeated US treatments performed weekly during the early phase (from disease onset/weight-peak to 10% weight loss) prolonged this early disease phase duration and increased survival.

### Motor neuron numbers were similar at the same disease stages among groups

**Fig. 4:**
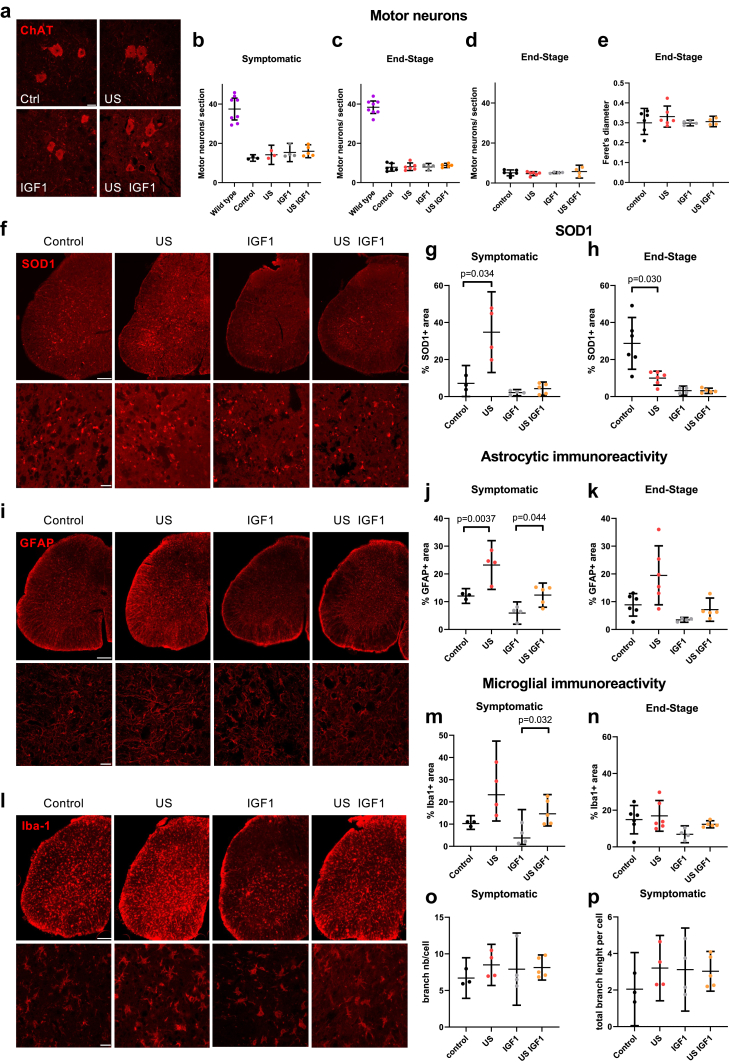
**Disruption of the blood spinal cord barrier (BSCB) by low intensity pulsed ultrasound (US) modifies glial cell immunoreactivity in the lumbar spinal cord of ALS mice.** (a) Images of ChAT-immunostained motor neurons in the lumbar spinal cord of ALS mice at disease end stage after the different treatments (ctrl: control, no treatment). (b–d) Motor neuron counts in the lumbar spinal cord after Nissl staining (b,c) at the symptomatic stage (symptomatic, b) or end stage (c), or after ChAT-immunostaining at disease end stage (d). For b, WT n = 8, control n = 3, US n = 3, IGF1 n = 4, US IGF1 n = 5; for c, WT n = 8, control n = 6, US n = 6, IGF1 n = 4, US IGF1 n = 5; for d, control n = 6, US n = 6, IGF1 n = 4, US IGF1 n = 4. (e) Motor neuron Feret’s diameter measured at disease end stage on ChAT-stained motor neurons. (f) Immunostaining against SOD1 of the lumbar spinal cords at the symptomatic stage in the different groups. (g and h) Quantification of the mutant SOD1+ immunoreactive area (symptomatic (g) control n = 3, US n = 4, IGF1 n = 4, US IGF1 n = 5, end stage (h) control n = 6, US n = 6, IGF1 n = 4, US IGF1 n = 5). p values were determined by Brown-Forsythe corrected ANOVA followed by Dunnett multiple comparison post hoc test. (i) Immunostaining against GFAP to stain astrocytes in the lumbar spinal cords at the symptomatic stage in the different groups. (j and k) GFAP immunoreactive area (symptomatic (j) control n = 3, US n = 4, IGF1 n = 4, US IGF1 n = 5, end stage (k) control n = 6, US n = 6, IGF1 n = 4 US IGF1 n = 5). p values were determined by ANOVA followed by a post hoc Šídák multiple comparison test. (l) Immunostaining against Iba1 to stain microglial cells in the lumbar spinal cords at the symptomatic stage in the different groups. (m and n) Iba1 immunoreactive area (symptomatic (m) control n = 3, US n = 4 IGF1 n = 4, US IGF1 n = 5, end stage (n) control n = 6, US n = 6, IGF1 n = 4, US IGF1 n = 5). p values were determined by Brown-Forsythe corrected ANOVA followed by Dunnett multiple comparison post hoc test. Bars represent mean with 95% CI. Scale bar: (a) 20 μm, (f, i, l) 200 μm for the upper panels, 20 μm for the lower panels. (o and p) Microglial morphology at the symptomatic stage measured on Iba1+ cells in lumbar spinal cords, control n = 3, US n = 4 IGF1 n = 4, US IGF1 n = 5. (o) Number of branches per microglial cell and (p) total branch length per microglia.

We aimed to analyse whether the improvement in the early disease phase duration in US-treated mice could also have led to increased preservation of motor neurons at the same disease stage. There was no difference in motor neuron counts among the different groups of ALS mice at the same disease stages of symptomatic stage or end stage (Fig. 4a–d) as well as no motor neuron soma size difference, measured on ChAT-stained motor neurons at disease end stage (Fig. 4a–e). However, one needs to keep in mind that due to the protective effect of the US treatment, US-treated ALS mice were older at the symptomatic stage (10% weight loss, end of the early phase, mean ± SD for US: 155.6 days ±9.3 and Control: 145 days ±10, p = 0.016; US IGF1: 152.7 days ±6.8 and IGF1: 142.1 days ±8.5, p = 0.0081, ANOVA followed by a post hoc Šídák multiple comparison test) and disease end stage than their respective controls (Fig. 3b), showing slower disease progression.

### Disease progression did not correlate with SOD1 burden

SOD1 burden (from the mutant human SOD1 transgene) was analysed at the symptomatic stage (defined by 10% weight loss) and end stage (with an antibody recognizing hSOD1 and its aggregates, Supplementary Fig. S3), to analyse potential short-term or long-term modifications of the SOD1 burden. As expected, the immunofluorescent area occupied by SOD1 in the spinal cord was higher in end stage ALS mice than in symptomatic mice (defined by 10% weight loss) (Fig. 4f–h). However, in symptomatic mice, the highest content of SOD1 among all groups was measured in mice in the US-treated group (Fig. 4f and g). In addition, both in symptomatic and end stage mice, the IGF1 group showed the lowest percentage of SOD1, which was comparable to that of mice in the US IGF1 group (Fig. 4f–h). Thus, as we did not find any correlation between SOD1 burden and the difference in survival measured after US treatment, we hypothesized that the mechanism by which US treatment prolonged survival was not linked to decreased mutant human SOD1 burden.

### US treatment in ALS mice modified glial cell immunoreactivity and molecular analysis revealed reduction of proliferative pathways in microglia

At the symptomatic stage, there was a difference in the immunoreactive area for GFAP (astrocyte staining) among the US and the control groups and among the US IGF1 and IGF1 groups (Fig. 4i–j). Mice that underwent US treatment showed higher astrocytic immunoreactivity than mice that did not undergo US treatment (Fig. 4i–j). US treatment alone led to the highest increase in astrocyte immunoreactivity (Fig. 4i–j), while IGF1 treatment alone led to a decrease compared to US IGF1 at the symptomatic stage (Fig. 4i–j). The effect of US treatment was not different at disease end stage with a spread in US-treated mice (Fig. 4k).

Microglial Iba1 immunoreactivity was measured in the different groups (Fig. 4l-p) and showed increased immunoreactive area after US treatment at the symptomatic stage (corresponding approximately to the end of the intervention) that was however different only between the US IGF1 and IGF1 groups and with a high spread in the US group (Fig. 4m).

To further analyse changes on the reactivity of microglial cells, we measured the morphology of their branching (number of branches and total branch length with ‘skeleton’ analysis) (Fig. 4o–p). Microglial morphology was not different between groups, however, potential finer morphological differences induced by US treatment could likely be masked by the overwhelming activation linked to ALS-mediated neurodegeneration.

**Fig. 5:**
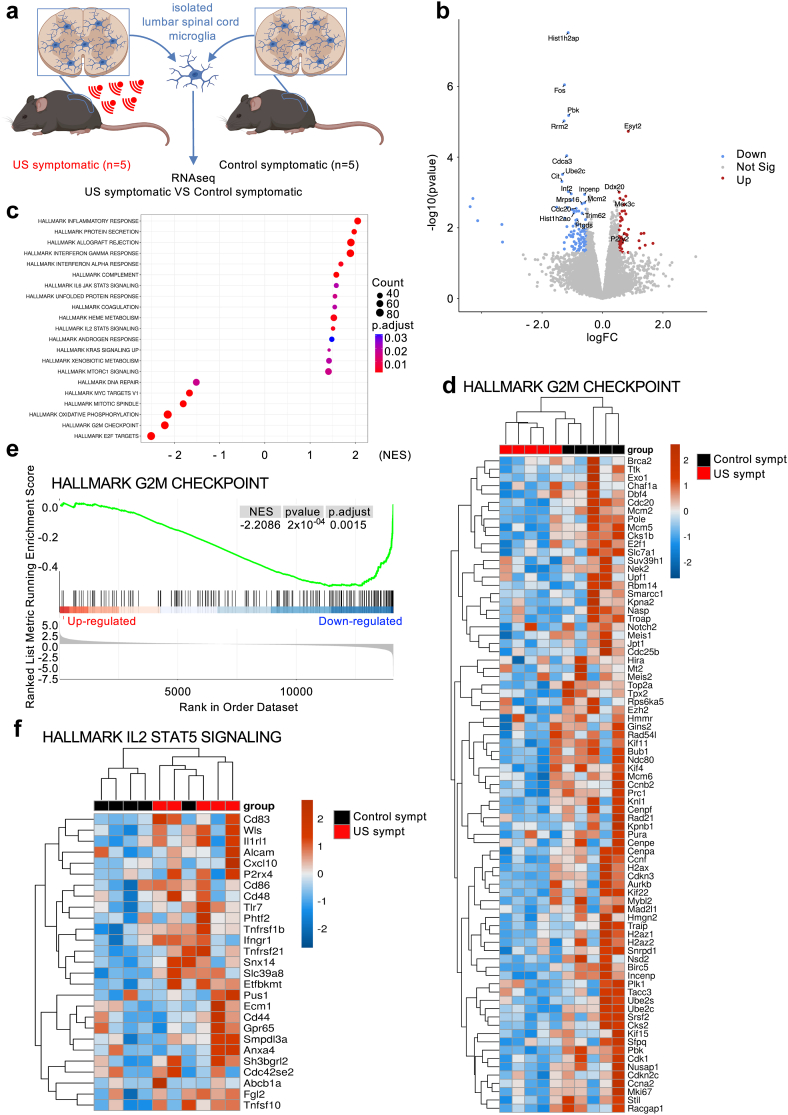
**Low intensity pulsed ultrasound (US) modifies microglial transcriptome in ALS mice.** (a) Experimental design for lumbar spinal cord isolated microglial cell transcriptome analysis of symptomatic ALS mice treated or not with US at the end of 5 repeated US treatment. Graphics done with Biorender. (b) Volcano plot of deregulated genes (FC > 1.5, p < 0.05; red: upregulated, blue: downregulated, grey: not regulated, Not Sig). (c) Enrichment analyses (‘Gene Set Enrichment Analysis’, GSEA) depicting significantly regulated pathways (p.adjust < 0.05, multiple hypothesis testing). Counts: number of genes in pathways, p.adjust: adjusted p value, NES: normalised enrichment score. (d) Heatmap of the Hallmark ‘G2M checkpoint’ pathway implicated in cell proliferation. (e) Gene Set Enrichment Analysis (GSEA) plot of the Hallmark ‘G2M checkpoint’ pathway. (f) Heatmap of the Hallmark ‘IL2 STAT5 signalling’ pathway implicated in regulatory T cell development, function and attraction. Heatmap scales in (d,f) are depicted as normalized read-counts by variance-stabilizing transformation (VST) centered on the mean for each gene.

We therefore analysed the transcriptome of microglial cells, isolated from the lumbar spinal cord of symptomatic ALS mice treated or not with US, at the end of the US treatment (Fig. 5a). RNAseq analyses of microglial cells from the US-treated group compared to the control group revealed 156 dysregulated genes (50 upregulated, 106 downregulated; FC > 1.5, p < 0.05). The most regulated genes were *Hist1h2ap* (H2A clustered histone 23), encoding a replication-dependant histone of the H2A family, and *Fos*, encoding the Fos proto-oncogene implicated as regulator of cell proliferation and differentiation, both downregulated in the US group compared to non-treated ALS controls (Fig. 5b). Enrichment analyses identified 21 significantly regulated pathways (Fig. 5c). The downregulated pathways were mainly linked to cell proliferation, including the Hallmark ‘G2M checkpoint’ (Fig. 5c–e), highlighting an impact of US treatment on diminishing microglial proliferation that usually occurs in symptomatic SOD1^G93A^ ALS mice. The upregulated pathways included pathways linked to inflammation including the Hallmark pathway ‘IL2 STAT5 signalling’ implicated in regulatory T cell development, function and attraction^41^ (Fig. 5c,f), and emphasising the interplay between microglia and lymphocytes.

### US treatment in ALS mice increased lymphocyte infiltration

**Fig. 6:**
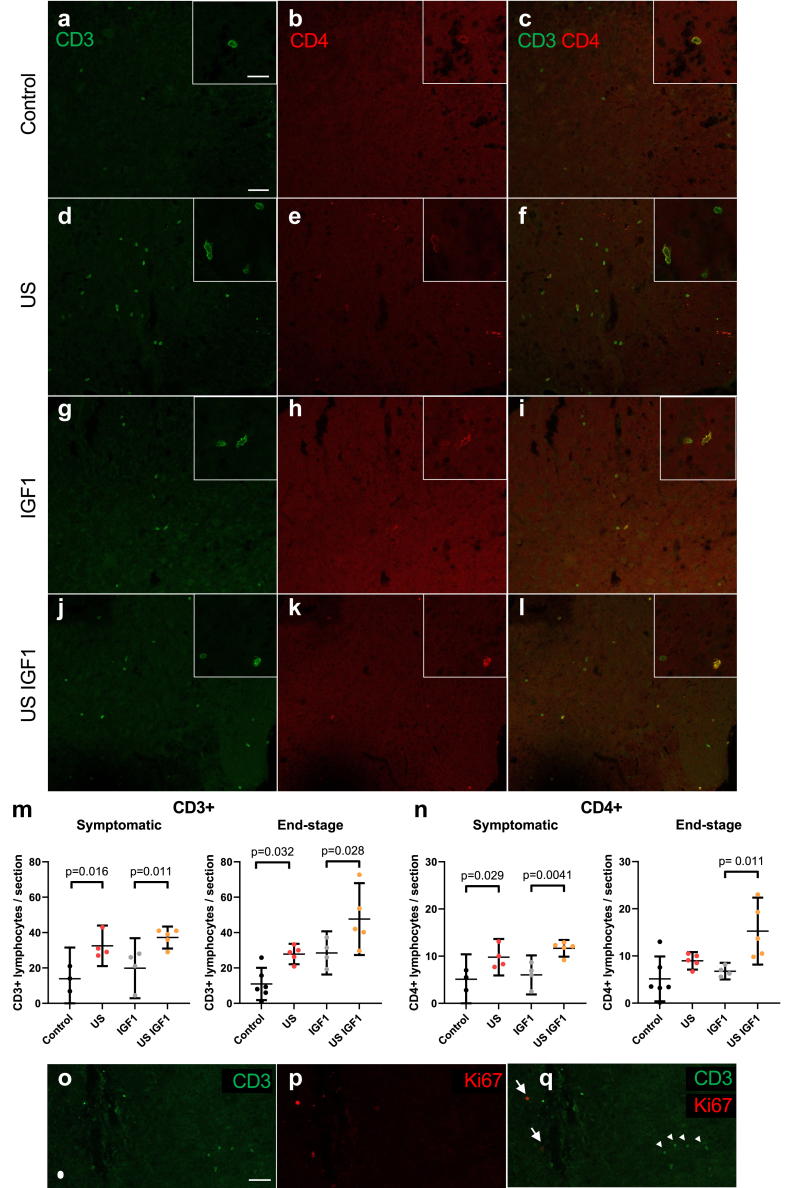
**Disruption of the blood spinal cord barrier (BSCB) by low intensity pulsed ultrasound (US) enhances CNS lymphocyte infiltration in ALS mice.** (a–l) Images of spinal cord sections of symptomatic stage ALS mice in the four different groups, immunostained for the lymphocyte pan-T CD3 (a,d,g,j, green) and specific lymphocyte T CD4 (b,e,h,k, red) markers and merged (c,f,i,l). Scale bars: 50 μm in (a) for (a–l) and 20 μm in inset of (a) for insets in (a–l). (m,n) Number of lymphocytes in the lumbar spinal cords of the different groups at the symptomatic stage (left graphs) or at disease end stage (right graphs), measured by counting the total number of CD3+ lymphocytes (m) or CD4+ cells (n) per spinal cord section (symptomatic, control n = 3, US n = 4, IGF1 n = 4, US IGF1 n = 5, end stage, control n = 6, US n = 5, IGF1 n = 4, US IGF1 n = 5). Bars represent mean with 95% CI. p values were determined by ANOVA followed by a post hoc Šídák multiple comparison test. (o–q) Images of a spinal cord section of a symptomatic stage ALS mouse that underwent US treatment, immunostained for the lymphocyte pan-T CD3 (o,q, green) and the cell proliferation marker Ki67 (p,q, red). Note that lymphocytes (arrowheads) are not Ki67 positive (arrows). Scale bar: 50 μm in (o) for (o–q).

Since our intervention transiently disrupted the BSCB, glial cell immunoreactivity was increased, microglial RNAseq analyses highlighted beside decreased proliferative pathways also increased pathways regulating T cells, and as CD4+ T cells are known to be protective in this ALS mouse model,^42^ we further examined the extent of lymphocyte infiltration in the different groups (Fig. 6a-n). At the symptomatic stage, there was a difference in the lymphocyte (CD3+) infiltration number, with a higher number of lymphocytes per section in mice that underwent US treatment than in mice that did not undergo US treatment (mean ± SD: US 32.5 ± 3.6, US IGF1 37 ± 5, control: 14 ± 4.1, IGF1: 20 ± 10.7, Fig. 6m). This difference persisted at the end stage as well (US: 28 ± 2.1, US IGF1: 48 ± 7.3, control: 11 ± 3.6, IGF1: 28.5 ± 3.8, Fig. 6m). CD4+ T-cell numbers were also quantified (Fig. 6n). At the symptomatic stage, the number of CD4+ T cells was higher in the US and US IGF1 groups than in their respective control groups (mean ± SD: US: 9.8 ± 2.4 vs. Control: 5.1 ± 2.1, US IGF1: 11.7 ± 1.4 vs. IGF1: 6 ± 2.6, Fig. 6n). This increased number of T cells was due to increased infiltration rather than due to proliferation as we only found very rare Ki67+/CD3+ cells (with Ki67 being a marker of proliferating cells, Fig. 6o-q) with 0.93% at the symptomatic stage and 0.33% at end stage. In addition to the findings of increased survival and prolonged early disease phase, these results suggest a beneficial effect of immune modulation from both US alone and US IGF1.

## Discussion

This study shows a surprising beneficial effect of US-mediated BSCB opening, by itself, on survival in a mutant SOD1 ALS mouse model. Treatments were started at the beginning of disease onset, not at a presymptomatic stage, to mimic a potential therapeutic intervention as would be performed in humans. Mice treated with five weekly US-based treatments to disrupt the BSCB, resulted in the longest increase in survival time of any of the treatment groups (+12 days), showing that this type of intervention, without any co-administered drug therapies, may be a promising therapeutic strategy for ALS. The 12-day increase in survival was associated with the prolongation of the early symptomatic phase of the disease (+10 days), during which the treatment was administered. A 12-day increase in survival in this ALS mouse model is similar to preclinical results reported with presymptomatic administration of riluzole, the current standard of care for ALS.^43^ This finding indicates that US treatment can delay clinical disease progression during the symptomatic phase in ALS mice, even if US treatment was only administered until the symptomatic stage and not until the end stage. Of note, we did not measure any difference in the grip-strength in the different groups. This could be due to the fact that the US procedure was performed in the lumbar region of the spinal cord while the grip-test we performed, more accurately measures the forelimb force. In addition, we did not measure a difference in the motor neuron cell number or soma size between groups, however, cell counts were performed at the same disease stages. Motor neuron loss was slower in mice in the US and US IGF1 groups than in mice in the control group, since similar motor neuron counts were obtained at an older age (+10 days) in mice that underwent US treatment. One also cannot rule out potential additional effects of US treatment on other systems including a vasogenic action on lower limb vessels.

IGF1 administration in combination with US treatment did not increase survival or functional tests. The lack of an additional benefit of IGF1 may be due to two reasons: an insufficient exogenous recombinant IGF1 administration or a sufficient endogenous mouse IGF1 infiltration in the spinal cord after US treatment. In our protocol, exogenous IGF1 was injected only once a week (matching US treatments when performed), for a total of five injections. This administration scheme differs significantly from published retrograde viral mediated IGF1 delivery schemes found to be effective in mouse models and leading to continuous exposure to IGF1.^21^ Additionally, in our study, the 0.5 mg/kg dose was an intermediate dose between the one used in the clinic (human clinical trial: 0.05 mg/kg, twice per day) and the one described as deleterious in this ALS mouse model with subcutaneous infusion (3 mg/kg).^44^ An explanation for the lack of additive effect on survival by US treatment with IGF1 injections, could be simply due to the possibility that the BSCB opening already allows enough other endogenous neurotrophic factors (like GDNF, BDNF^45^ or IGF1 itself) to pass into the CNS and the additional IGF1 injection did not further enhance this effect.

Unexpectedly, at the symptomatic stage (after US treatment), we observed an increase in mutant SOD1 immunoreactivity in the CNS of mice that underwent US treatment. SOD1 is ubiquitously expressed in SOD1^G93A^ ALS mice, and we could not assess whether the protein was more endogenously expressed by cells in the CNS or whether it originated from the periphery and was released in the CNS after BSCB opening. On the other hand, at the end stage, when mutant SOD1 protein increased in the spinal cord of ALS mice compared to the symptomatic stage, we observed a lower quantity of mutant SOD1 immunoreactivity in the CNS of ALS mice that underwent US treatment, suggesting a potential clearance phenomenon. Such clearance of intracellular inclusions after US treatment has already been observed for the tau protein in Alzheimer’s disease models.^46^ The mechanism for intracellular protein clearance remains elusive, but it is interesting to note that it occurs concomitantly with immune activation. Since mutated SOD1 causes neurodegeneration through a toxic gain-of-function, it is possible that the reduction in SOD1 quantity we observed after this transient increase partially explains the prolonged survival of mice that underwent US treatment. However, since reduction of SOD1 immunoreactivity was also measured in mice treated with IGF1 alone, increased survival after US treatment cannot be explained by reduced SOD1 burden in the spinal cord.

T-lymphocytes are known to play a protective role in ALS mice, as crossing SOD1^G93A^ mice with Rag2**^−^^−^**^^-^^ mice (which do not produce any lymphocytes) accelerates disease progression.^42^ This protective effect was associated with CD4+ T lymphocytes,^42^^,^^47^^,^^48^ while reduction of CD8+ T lymphocytes (SOD1^G93A^ mice crossed with CD8**^−^**^/^**^−^** mice or anti-CD8+ blocking antibodies) did not impact ALS mouse survival.^49^^,^^50^ In addition, a lower proportion of CD4+ T lymphocytes was associated with fast progression in patients with ALS.^48^^,^^51^ Interestingly, in our study, we observed that US treatment in ALS mice increased lymphocyte CNS infiltration with an overall increase in the number of lymphocytes, including CD4+ T lymphocytes. Thus, one hypothesis would be that infiltration of CD4+ T lymphocytes from the periphery to the CNS could modify the inflammatory responses of resident glial cells as well as increase neuronal survival. Of note, ALS mice lacking CD4+ T cells survived less long, and interestingly, also had reduced IGF1 levels in the spinal cord.^42^ In addition, our microglial transcriptome analysis comparing US-treated and control ALS mice showed an increased pathway implicated in lymphocyte attraction and differentiation, which corroborates a possible interaction between microglia and lymphocytes in US-treated ALS mice.

An increase in glial cell activation by US-based BBB opening has already been observed in preclinical studies.^52^ Localized BBB opening in nonhuman primates showed that US-treatment triggers a short-lived immune response within the targeted region with increased microglial density around blood vessels detected on day 2 and resolved by day 18.^53^ US also attenuated the proinflammatory responses in microglia induced by lipopolysaccharide (LPS).^54^ A beneficial effect of US alone has already been observed in Alzheimer's disease both behaviourally and histologically in terms of a reduction in amyloid plaques and tau protein.^55^, ^56^, ^57^ In our study, US treatment also induced increased activation of glial cells (microglia and astrocytes), measured by immunoreactive area, in response to opening the BSCB. Microglial activation plays a complex role in the progression of ALS and can have both protective and/or neurotoxic consequences.^29^^,^^42^^,^^58^^,^^59^ In our microglial transcriptome study, although several pathways implicated in inflammation were upregulated after US treatment, they included both genes with rather pro- or anti-inflammatory potential and included the anti-inflammatory Hallmark “IL2 STAT5 signalling” pathway (linked to regulatory T cell development, function and attraction). In addition, microglial cells of US-treated ALS mice showed decreased expression of genes and pathways implicated in cell proliferation. Previous studies have analysed the impact of US on homeostatic microglia and showed an increase in cell proliferation.^60^^,^^61^ Since we measure the opposite in ALS mice, US could have a different impact on microglial cells that are already activated and proliferating in a response to ongoing neurodegeneration.

As a limitation of our study, experiments were performed in female mice only, which restrains generalizability to males. In addition, the SOD1^G93A^ ALS mouse model used in this study is a particularly severe ALS model, in which mice quickly become too weak to tolerate general anesthesia. This led us to stop treatments at 135 days of age and thus allowed for only a short window of five weeks (5 sessions) for therapeutic intervention. Therefore, the potential benefit of prolonged exposure to US procedures could not be assessed, as these mice were unable to tolerate prolonged treatments during the late phase of their disease. Further studies will focus on the coadministration of other drugs to amplify the beneficial effects observed of US treatment alone.

In summary, this study shows a beneficial effect of US mediated BSCB opening alone, even without any additional drug therapy, on survival in an aggressive mutant SOD1 ALS mouse model. This shows that the US technique could be a promising therapeutic strategy for treatment of ALS and other CNS disorders.

## Contributors

All authors read and approved the final version of the manuscript.

Anne-Sophie Montero and Séverine Boillée have accessed and verified the underlying data.

**Anne-Sophie Montero**: conceptualization, formal analysis, investigation, project administration, visualisation, writing—original draft.

**Ilyes Aliouat**: formal analysis, investigation, visualisation, writing—review & editing.

**Matthieu Ribon**: investigation, visualization, writing—review & editing.

**Michael Canney**: methodology, visualization, writing—review & editing.

**Lauriane Goldwirt**: investigation.

**Samia Mourah**: investigation.

**Félix Berriat**: investigation, writing—review & editing.

**Christian S Lobsiger**: investigation, methodology, writing—review & editing.

**Pierre-François Pradat**: writing—review & editing.

**François Salachas**: writing—review & editing.

**Gaëlle Bruneteau**: writing—review & editing.

**Alexandre Carpentier**: conceptualization, funding acquisition, methodology, project administration, supervision, writing—review & editing.

**Séverine Boillée**: conceptualization, funding acquisition, methodology, resources, project administration, supervision, writing—review & editing.

## Data sharing statement

All data generated during this study are available in the main text. Raw RNAseq data are accessible on NCBI GEO under the accession code GSE269925.

## Declaration of interests

Michael Canney is an employee of Carthera and has ownership interest in the company as well as patents related to this technology. Alexandre Carpentier is a consultant to Carthera, has ownership interest in the company, and has filed patents pertaining to the results presented in the paper. Gaelle Bruneteau: Grants from the French association for ALS (ARSLA) and the French association for Myopathies (AFM), National Hospital Clinical Research Programs (PHRC-N).
