# Supplementary material for: Effect of ultrasound-mediated blood-spinal cord barrier opening on survival and motor function in females in an amyotrophic lateral sclerosis mouse model
Source: eBioMedicine. 2024 Jul 13;106:105235. doi: 10.1016/j.ebiom.2024.105235 (PMC11284947; doi:10.1016/j.ebiom.2024.105235)
Supplement: Supplementary Figs. S1–S3 and Tables S1 and S2 [file mmc1.pdf]

Effect of ultrasound-mediated blood-spinal cord barrier opening on survival and motor function in females in an Amyotrophic Lateral Sclerosis mouse model

Authors: Anne-Sophie Montero[#1,2,3,4,5], Ilyes Aliouat[#4], Matthieu Ribon[4], Michael Canney[6], Lauriane Goldwirt[7], Samia Mourah[7], Félix Berriat[4], Christian S. Lobsiger[4], Pierre-François Pradat[8], François Salachas[4,8], Gaëlle Bruneteau[8], Alexandre Carpentier[1,2,3], Séverine Boillée[4*]

Supplementary Table 1: Supplementary Table 1. Reporting of mean or median differences with 95% CI and p values. .

Supplementary Table 2: Summary of complications of the procedure

Supplementary Figure 1: Grip test measures for the mouse cohorts.

Supplementary Figure 2: Body weight measures for the mouse cohorts.

Supplementary Figure 3: Anti-SOD1 staining shows aggregated structures in SOD1G93A mouse spinal cords.

	Median differences	95% CI	P value
**Figure 2b**			
EBD VS US EBD	-67	-127 to -28	0.00060
IGF1 VS US IGF1	-172	-265.0 to -100.8	<0.0001

	Mean differences	95% CI	Adjusted P value
**Figure 3c**			
Control vs. US	-10.4	-20.25 to -0.5525	0.037
IGF1 vs. US IGF1	-10.33	-19.32 to -1.344	0.022
**Figure 3d**			
Control vs. US	0.5	-8.423 to 9.423	0.99
IGF1 vs. US IGF1	-0.8667	-9.013 to 7.279	0.96
**Figure 4b**			
Control vs. US	-1.6	-8.306 to 5.106	0.48
IGF1 vs. US IGF1	-0.63	-6.113 to 4.853	0.93
**Figure 4c**			
Control vs. US	-0.2833	-3.098 to 2.531	0.96
IGF1 vs. US IGF1	-0.77	-2.796 to 1.256	0.50
**Figure 4d**			
control vs. US	0.3898	-1.458 to 2.237	0.85
IGF1 vs. US IGF1	-0.5134	-2.776 to 1.749	0.83
**Figure 4e**			
control vs. US	-0.02622	-0.1077 to 0.05527	0.65
IGF1 vs. US IGF1	-0.007231	-0.03650 to 0.02204	0.71
**Figure 4g**			
Control vs. US	-27.64	-52.01 to -3.256	0.034
IGF1 vs. US IGF1	-2.166	-6.456 to 2.124	0.31
**Figure 4h**			
Control vs. US	18.77	2.323 to 35.21	0.030
IGF1 vs. US IGF1	0.075	-2.797 to 2.947	0.99
**Figure 4j**			
Control vs. US	-11.14	-18.31 to -3.980	0.0037
IGF1 vs. US IGF1	-6.465	-12.76 to -0.1729	0.044
**Figure 4k**			
Control vs. US	-10.63	-23.53 to 2.271	0.098
IGF1 vs. US IGF1	-3.693	-8.882 to 1.496	0.13
**Figure 4m**			
Control vs. US	-13.7	-34.46 to 7.055	0.14
IGF1 vs. US IGF1	-10.4	-19.74 to -1.062	0.032
**Figure 4n**			
Control vs. US	-2.047	-10.62 to 6.526	0.81
IGF1 vs. US IGF1	-5.434	-15.39 to 4.527	0.36
**Figure 4o**			
control vs. US	-1.801	-5.713 to 2.111	0.46
IGF1 vs. US IGF1	-0.2266	-3.663 to 3.209	0.98
**Figure 4p**			
Ctrl vs. US	-1.152	-3.278 to 0.9746	0.35
IGF1 vs. US IGF1	0.09237	-1.775 to 1.960	0.99
**Figure 6m symptomatic**			
Control vs. US	-18.57	-33.44 to -3.697	0.016
IGF1 vs. US IGF1	-17.33	-30.39 to -4.265	0.011
**Figure 6m End-stage**			
Control vs. US	-16.89	-32.35 to -1.438	0.032
IGF1 vs. US IGF1	-19.14	-36.26 to -2.019	0.028
**Figure 6n Symptomatic**			
Control vs. US	-4.675	-8.869 to -0.4812	0.029
IGF1 vs. US IGF1	-5.635	-9.318 to -1.952	0.0041
**Figure 6n End-stage**			
Control vs. US	-3.827	-9.687 to 2.034	0.24
IGF1 vs. US IGF1	-8.485	-14.98 to -1.993	0.011

Supplementary Table 1. Reporting of mean or median differences with 95% CI and p values.

	control	US	IGFI	IGFI US	Total
Nb of procedures	**70**	**117**	**71**	**125**	**383**
Nb of mice	15	25	15	25	80
procedure death rate *	**2 (2.9%)**	**5 (4.3%)**	**1 (1.4%)**	**0 (0%)**	**8 (2.1%)**
Toxicity US		**1 death**<br>*paraplegia*<br>**1 deficit** reversible		**1 deficit** partially reversible<br>**1 deficit** reversible	**1 death**<br>1 persistant deficit<br>2 reversible déficits
Toxicity of injections	**2 deaths**	**2 deaths**	**1 death**		**5 deaths**
other complications		**2 deaths**<br>*hyperthermia*			**2 deaths**

Supplementary Table 2. Summary of complications of the procedure. * procedure death rate, calculated per procedure, as deficit or death were occurring immediately after the procedure of ultrasound (US) or retro-orbital injections.

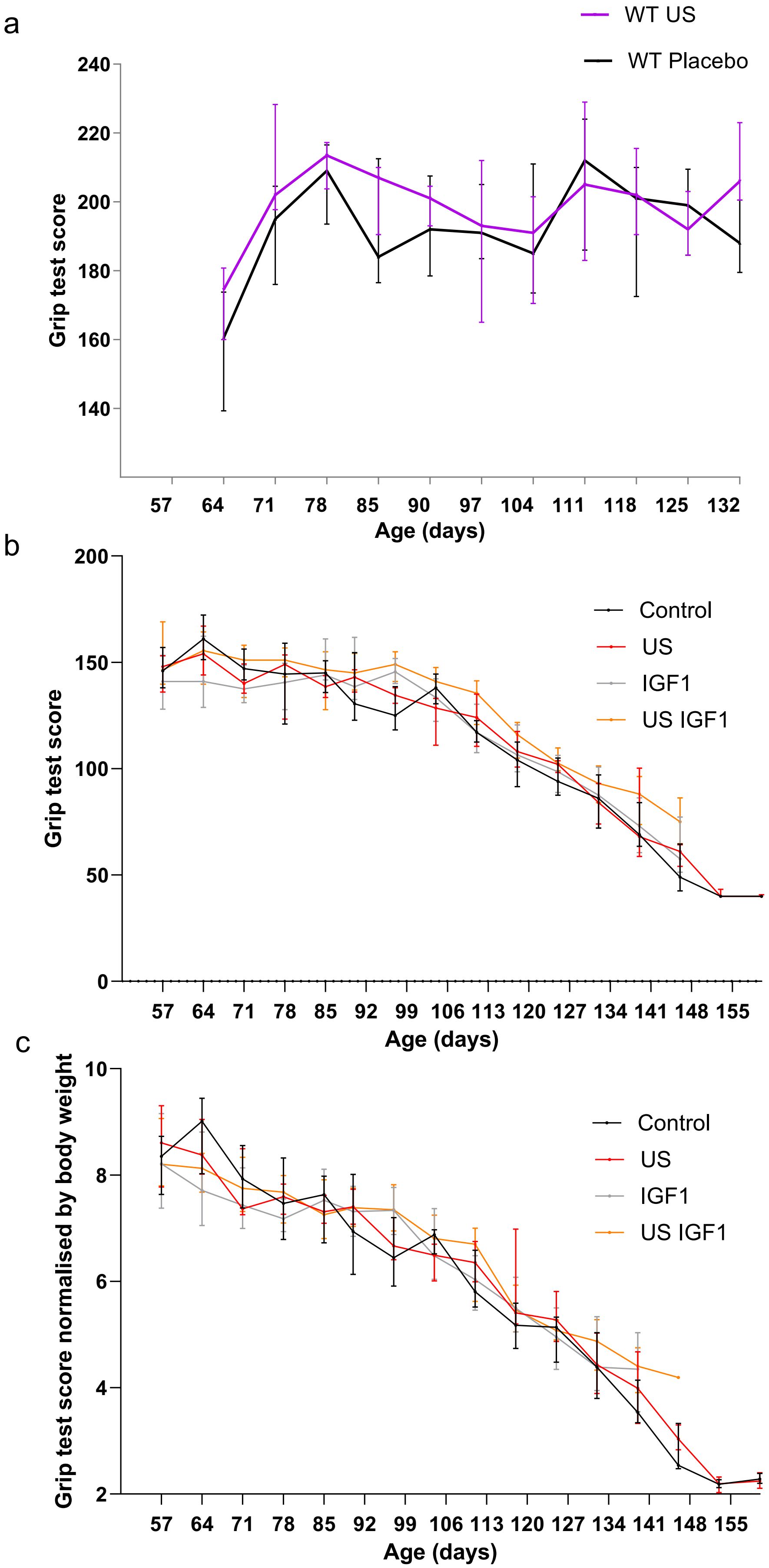

Supplementary Figure 1. Grip test measures for the mouse cohorts. Muscle strength was measured using the grip test in (a) wild-type (WT) mice used to measure safety and efficacy of BSCB opening after US treatment (WT US, n=10) or without US treatment (WT Placebo, n=10) starting at the age of 9 weeks, and (b) starting at the age of 8 weeks, in the 4 groups of $SOD1^{G93A}$ ALS mice that either did not receive any treatment (control, n=10), or received US treatment (US, n=10), subcutaneous IGF1 (IGF1, n=10) or IGF1 followed by US treatment (US IGF1, n=15). (c) Grip test scores normalized by body weight (control n=10, US n=10, IGF1 n=10, US IGF1, n=15). Curves represent Median and interquartile range.

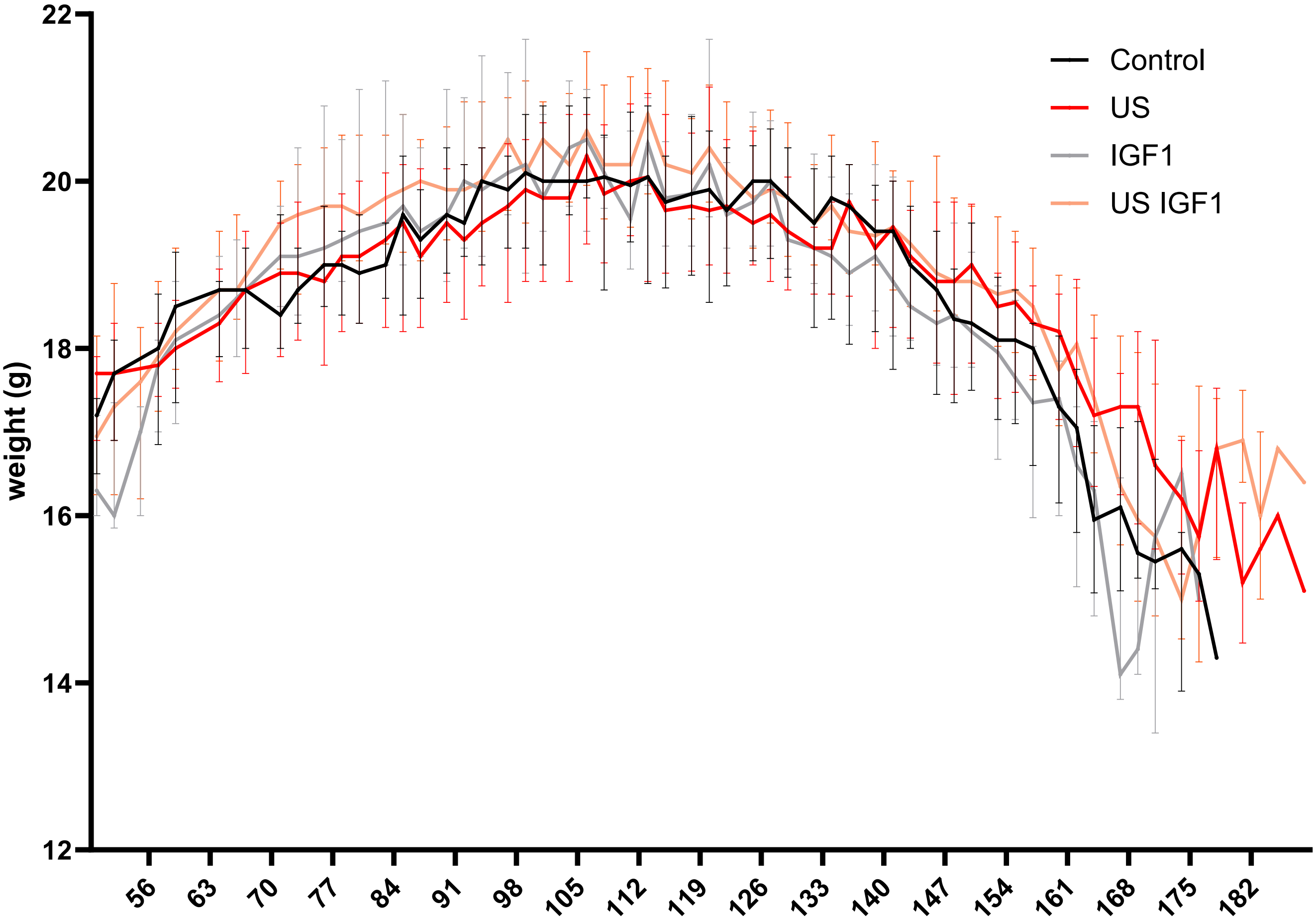

Supplementary Figure 2. Body weight measures for the mouse cohorts. Body weight was measured once a week starting at the age of 7 weeks for the whole duration of the follow-up (control n=10, US n=10, IGF1 n=10, US IGF1, n=15). Curves represent Median and interquartile range.

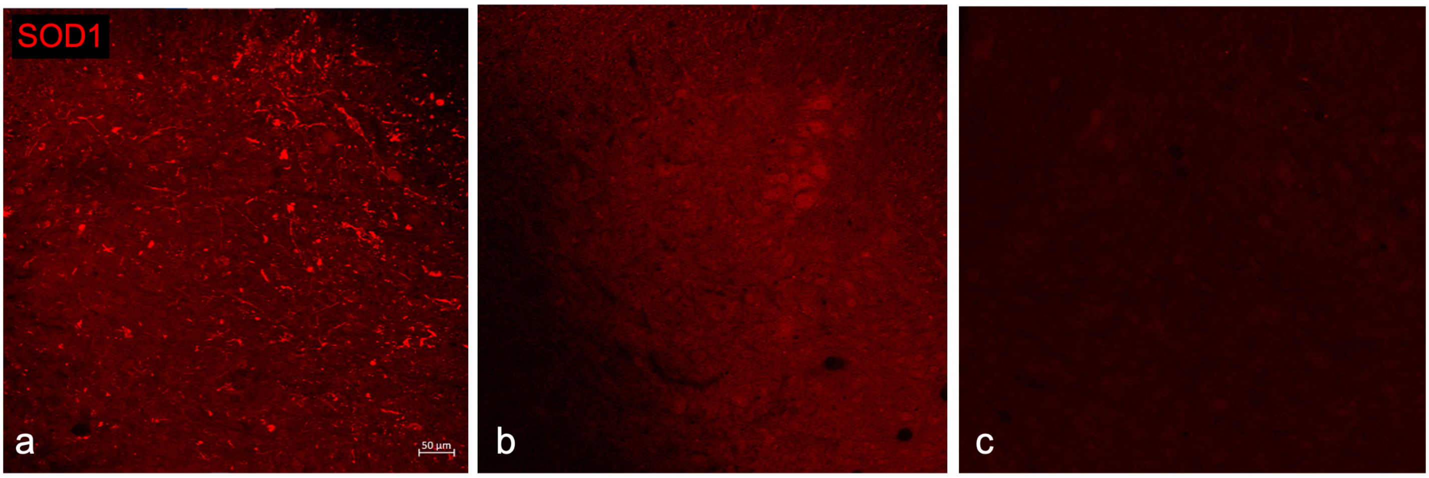

Supplementary Figure 3. Anti-SOD1 staining shows aggregated structures in SOD1^{G93A} mouse spinal cords. Immunostaining against SOD1 in (a) SOD1^{G93A}, (b) transgenic hSOD1^{WT} and (c) C57Bl6/J mouse spinal cord sections. Scale bar: 50µm.
